# Gastrectomy for stage IV gastric cancer: a comparison of different treatment strategies from the SEER database

**DOI:** 10.1038/s41598-021-86352-6

**Published:** 2021-03-30

**Authors:** Jacopo Desiderio, Andrea Sagnotta, Irene Terrenato, Bruno Annibale, Stefano Trastulli, Federico Tozzi, Vito D’Andrea, Sergio Bracarda, Eleonora Garofoli, Yuman Fong, Yanghee Woo, Amilcare Parisi

**Affiliations:** 1 Department of Digestive Surgery, Azienda Ospedaliera Santa Maria, Via Tristano di Joannuccio 1, 05100 Terni, Italy; 2 Department of Surgical Sciences – PhD Program in Advanced Surgical Technologies, Sapienza University of Rome, Rome, Italy; 3 Department of General Surgery and Surgical Oncology, San Filippo Neri Hospital, Rome, Italy; 4 Department of Medical-Surgical Sciences and Translational Medicine, Sant’Andrea Hospital, Sapienza University of Rome, Rome, Italy; 5 Biostatistics and Bioinformatic Unit, Scientific Direction, IRCCS Regina Elena National Cancer Institute, Rome, Italy; 6 Division of Surgical Oncology and Endocrine Surgery, Mays Cancer Center, University of Texas Health Science Center San Antonio, San Antonio, TX USA; 7 Medical and Translational Oncology, Department of Oncology, Azienda Ospedaliera Santa Maria, Terni, Italy; 8 Division of Surgical Oncology, Department of Surgery, City of Hope National Medical Center, Duarte, CA USA

**Keywords:** Gastric cancer, Surgical oncology

## Abstract

In the West, more than one third of newly diagnosed subjects show metastatic disease in gastric cancer (mGC) with few care options available. Gastrectomy has recently become a subject of debate, with some evidence showing advantages in survival beyond the sole purpose of treatment tumor-related complications. We investigated the survival benefit of different strategies in mGC patients, focusing on the role and timing of gastrectomy. Data were extracted from the SEER database. Groups were determined according to whether patients received gastrectomy, chemotherapy, supportive care. Patients receiving a multimodality treatment were further divided according to timing of surgery, whether performed before (primary gastrectomy, PG) or after chemotherapy (secondary gastrectomy, SG). 16,596 patients were included. Median OS was significantly higher (*p* < 0.001) in the SG (15 months) than in the PG (13 months), gastrectomy alone (6 months), and chemotherapy (7 months) groups. In the multivariate analysis, SG showed better OS (HR = 0.22, 95%CI = 0.18–0.26, *p* < 0.001) than PG (HR = 0.25, 95%CI = 0.23–0.28, *p* < 0.001), gastrectomy (HR = 0.40, 95%CI = 0.36–0.44, *p* < 0.001), and chemotherapy (HR = 0.42, 95%CI = 0.4–0.44, *p* < 0.001). The survival benefits persisted even after the PSM analysis. This study shows survival advantages of gastrectomy as multimodality strategy after chemotherapy. In selected patients, SG can be proposed to improve the management of stage IV disease.

## Introduction

Gastric cancer (GC) is the third leading cause of cancer-related deaths worldwide, with 783,000 GC deaths in 2018^1, 2^. Stage IV GC is considered terminal, with survival rarely exceeding one year. Palliative management seeks to control disease progression and relieve GC-related symptoms. While various combinations of chemotherapeutic agents, radiation therapy, and endoscopic and surgical interventions with supportive care have been shown to increase survival compared to supportive care alone^3^, there is little improvement in the long-term survival rate of these patients.

The optimal strategy remains unclear amid a lack of scientific evidence and variability among possible management approaches. In most patients, treatment is often proposed to address needs as they arise rather than as part of a planned tailored treatment pathway. In the West, more than a third of patients with GC are diagnosed with metastatic disease (stage IV) at the time of the initial clinical evaluation, a trend that poses a complex management challenge. Traditionally, surgery has not been considered a therapeutic option for stage IV GC, except in patients with symptoms such as bleeding, perforation, or obstruction, who may require urgent operations. For other patients who do not require urgent intervention at diagnosis but are at high risk of related GC-complications that may require surgical intervention, the role of gastrectomy has recently become a major subject of debate. Moreover, as improvements in surgical techniques and supportive care measures allow patients a safer and more rapid recovery from surgery with lower morbility and mortality (0–5%) rates than in the past^4^, the role of surgery in stage IV GC is constantly being reexamined.

The rationale for gastric resection for stage IV patients finds its principle in the WHO statement for palliative care: “improve the quality of life of the patient through the prevention and relief of suffering”^5^. Therefore, the primary objective of the palliative surgery involves alleviating cancer-related symptoms and preventing the tumor’s otherwise inevitable complications. Furthermore, some authors hypothesize that removing the primary tumor and thus reducing the tumor burden could improve survival, as seen in other tumor types^6, 7^. A heterogeneous group of recent studies have emphasized that gastrectomy may achieve better symptom control^8^, improved quality of life^9^, and even in some patients, increase overall survival^10^. To date, however, the role and timing of gastrectomy in the non-urgent palliative setting have not been well clarified and a recent randomized trial (REGATTA^11^) demonstrated that the initial removal of the primary tumor is not necessarily beneficial. On the other hand, other authors^12^ have successfully highlighted a possible role for gastrectomy with radical intent after induction chemotherapy (conversion surgery).

We conducted a large population-based study to investigate the survival benefits of different treatment strategies focusing on the role and timing of gastrectomy. We further propose strategies for the optimal management of stage IV patients by combining our findings with the best evidence from the current literature.

## Materials and methods

### Patients source and definitions

Eligible patients were identified from the Surveillance, Epidemiology, and End Results (SEER) database^13^. Detailed data were obtained by the SEER-stat software (SEER*Stat 8.3.5). The following patients were included in the analysis: aged 18 years or older, diagnosis of stage IV GC (Primary Site-labeled: C16.1-Fundus of stomach, C16.2-Body of stomach, C16.3-Gastric antrum, C16.4-Pylorus, C16.5-Lesser curvature of stomach NOS, C16.6-Greater curvature of stomach NOS, C16.8-Overlapping lesion of stomach, C16.9-Stomach, NOS) according to the *AJCC Staging System*, 8th edition^14^. Histology was confirmed using the International Classification of Disease for Oncology (ICD-O-3; M-8010/3 through M-8015/3, M-8020/3 through M-8022/3, M-8030/3 through M-8035/3, M-8041/3, M-8043/3, M-8050/3 through M-8052/3, M-8070/3 through M-8078/3, M-8140/3 through M-8145/3, M-8147/3, M-8210/3 through M-8211/3, M-8214/3, M-8220/3, M-8221/3, M-8230/3, M-8231/3, M-8255/3, M-8260/3 through M-8263/3, M-8310/3, M-8323/3, M-8480/3, M-8481/3, M-8490/3, M-8510/3, M-8560/3, M-8562/3, M-8570/3 through M-8576/3, M-8980/3 through M-8982/3). We excluded patients with cardias tumors (C16.0-Cardia NOS) and those who lacked adequate information on treatment and follow-up duration.

### Decoding of treatments

The eligible population was classified according to whether the patients received primary cancer resection via the site-specific surgery of primary site codes. The surgery group was divided into total (or near-total) gastrectomy (codes 40–42, 50, 52, 62) and partial gastrectomy (codes 30–33, 51, 60, 61, 63). The “gastrectomy performed,” “CHT recode,” and “radiation recode” codes were used to explore if single or multiple treatments were administrated. Finally, the “CS Tumor Size/Ext Eval (2004 +)” and “CS Reg Node Eval (2004 +)” codes allowed patient classification according to timing of surgery performed before (primary gastrectomy [PG]) or after CHT (secondary gastrectomy [SG]). Patients not included in the other treatment categories were considered in the best supportive care (BSC) group.

The variable “radical intent” (yes/no) combines performing a gastrectomy with or without extensive lymphadenectomy and/or removing lesions beyond the primary site (“RX Summ–Surg Oth Reg/Dis”). The variable “Response to NAT” (regression, stable, progression) evaluates staging changes in patients who underwent SG combining “CS Lymph nodes” and “CS Site-Specific Factor 1”. Patients defined as “Responders” are those in whom treatments have achieved a prolonged overall survival over a period of 6 months.

“Performance status,” intended to assign patients to a “good” or “poor” category, was estimated using the claims-based measures described in previous reported models^15–17^. The “complicated disease” (yes/no) variable stratifies patients considering a severe disease presentation based on an obstructive mass description and/ or invasion of vital organs.

### Statistical analysis

Descriptive statistics were used to summarize pertinent study information and categorical data were compared by the the χ2 test. Overall survival (OS) was defined as the duration from the date of diagnosis to death or last follow-up, with no restriction on the cause of death. Cancer-specific survival (CSS) was defined as the duration from the date of diagnosis until death due to gastric cancer other than other causes. Patients with a follow-up < 1 month and without data about alive or dead status were excluded from survival analyses. OS and CSS were calculated by the Kaplan–Meier product-limit method. The log-rank test was used to assess differences between subgroups. The Hazard Risk and its relative 95% confidence interval (95%CI) was estimated for each variable using the Cox proportional univariate model adopting the most suitable prognostic category as referent group. Multivariate Cox proportional hazard model was also developed using stepwise regression (forward selection). Enter limit and remove limit were *p* = 0.05 and *p* = 0.10, respectively. Significance was defined at the *p* = 0.05 level. In order to control for potential confounders that could affect the outcomes of interest, propensity score matching (PSM)^18, 19^ was employed to generate two different treatment groups with balanced distribution of baseline features. Propensity scores resulting from logistic regression with dependent variable being the choice of undergo surgery (Primary surgery was considered as control practice). Included covariates were age at diagnosis, gender, race, primary site, N status and type of metastases at diagnosis. Patients were matched 1:1 with the nearest-neighbor method using a caliper distance of 0.15 of the standard deviation of the logit of the estimated propensity score to ensure good matches. Balance between the two groups was assessed using the relative multivariate imbalance measure L1 proposed by Iacus, King and Porro^20, 21^.

In order to compare CHT vs SG we analyzed the subgroup of patients considerable as “responders” to each specific treatment. We conducted another propensity score mathcing resulting from logistic regression with dependent variable being the choice of undergo surgery (CHT only was considered as control practice). Included covariates included were age at diagnosis, gender, race, primary site and type of metastases at diagnosis. Patients were matched 1:1 with the nearest-neighbor method using no caliper distance of the standard deviation of the logit of the estimated propensity score to ensure good matches. Balance between the two groups was assessed using the relative multivariate imbalance measure L1 proposed by Iacus, King and Porro^20, 21^.

All analyses were carried out with SPSS (21.0).

## Results

### Patients baseline characteristics (overall sample)

According to our inclusion criteria, 16,596 patients with stage IV gastric carcinoma at diagnosis between 2004 and 2015 were included. There were 9454 males (57%), and the mean age was 65 ± 15 years. In the majority of patients, 4026 patients (24.2%), the tumor was located at the antrum/pylorus, and a signet ring cell adenocarcinoma was reported in 4387 patients (26.4%). GC was most frequently poorly differentiated (n = 9925; 59.8%), with an advanced T stage (T3-T4 n = 6665; 67.5%). A total of 12,421 patients (74.8%) presented with distant metastases at diagnosis, 1478 (8.9%) with distant pathological lymphnodes, and 2697 (16.3%) with evidence of both distant pathological lymphnodes and metastases. Detailed clinicopathologic characteristics and related division by treatments are reported in Table 1.

**Table 1:** Baseline characteristics of patients with stage IV GC.

Characteristics	Total (%) N = 16,596	BSC (%) N = 7282	CHT + / − RT (%) N = 6819	Gastrectomy (%) N = 1244	Primary Gastrectomy (%) N = 1031	Secondary Gastrectomy (%) N = 220	*p*
**Year of diagnosis**							< **0.0001**
2004–2006	4037 (24.3)	1917 (26.3)	1287 (18.9)	454 (36.5)	355 (34.4)	24 (10.9)	
2007–2010	5493 (33.1)	2402 (33)	2206 (32.4)	435 (35)	384 (37.2)	66 (30)	
2011–2015	7066 (42.6)	2963 (40.7)	3326 (48.8)	355 (28.5	292 (28.3)	130 (59.1)	
**Sex**							0.083
Male	9454 (57)	4149 (57)	3907 (57.3)	701 (56.4)	592 (57.4)	105 (47.7)	
Female	7142 (43)	3133 (43)	2912 (42.7)	543 (43.6)	439 (42.6)	115 (52.3)	
**Age**							< **0.0001**
< 65	7646 (46.1)	2503 (34.4)	3948 (57.9)	413 (33.2)	630 (61.1)	152 (69.1)	
≥ 65	8950 (53.9)	4779 (65.6)	2871 (42.1)	831 (66.8)	401 (38.9)	68 (30.9)	
**Race**							
White	11,108 (67)	4865 (67)	4664 (68.7)	803 (64.6)	637 (61.9)	139 (63.8)	< **0.0001**
Black	2684 (16.3)	1233 (17)	1072 (15.8)	191 (15.4)	157 (15.3)	31 (14.2)	
Other	2748 (16.7)	1160 (16)	1056 (15.5)	249 (20)	235 (22.8)	48 (22)	
**Marital status**							< **0.0001**
Unmarried	9208 (55.4)	3506 (48.1)	4201 (61.6)	696 (55.9)	661 (64.1)	144 (65.5)	
Married	6681 (40.3)	3436 (47.2)	2347 (34.4)	497 (40)	339 (32.9)	62 (28.2)	
Unknown	707 (4.3)	340 (4.7)	271 (4)	51 (4.1)	31 (3)	14 (6.4)	
**Insurance status**							< **0.0001**
Insured	11,533 (69.5)	4866 (66.8)	5122 (75.1)	733 (58.9)	627 (60.8)	185 (84.1)	
Uninsured	4767 (28.7)	2253 (30.9)	1599 (23.4)	490 (39.4)	392 (38)	33 (15)	
Unknown	296 (1.8)	163 (2.2)	98 (1.4)	21 (1.7)	12 (1.2)	2 (0.9	
**Performance status**							< **0.0001**
Good	10,212 (61.5)	3567 (49)	5024 (73.7)	639 (51.4)	797 (77.3)	185 (84.1)	
Poor	6384 (38.5)	3715 (51)	1795 (26.3)	605 (48.6)	234 (22.7)	35 (15.9)	
**Complicated disease**							< **0.0001**
No	5748 (34.6)	2007 (27.6)	2235 (32.8)	743 (59.7)	630 (61.1)	133 (60.5)	
Yes	10,848 (65.4)	5275 (72.4)	4584 (67.2)	501 (40.3)	401 (38.9)	87 (39.5)	
**Site of tumor**							< **0.0001**
Fundus-Body	3345 (20.2)	1440 (19.8)	1537 (22.5)	167 (13.4)	146 (14.2)	55 (25)	
Antrum-Pylorus	4026 (24.2)	1651 (22.7)	1440 (21.1)	481 (38.7)	400 (38.8)	54 (24.5)	
Overlapping lesion of the stomach	2191 (13.2)	862 (11.8)	962 (14.)	170 (13.7)	147 (14.3)	50 (22.7)	
Stomach, NOS	7034 (42.4)	3329 (45.7)	2880 (42.2)	426 (34.1)	338 (32.8)	61 (27.7)	
**Histology**							< **0.0001**
Adenocarcinoma/carcinoma, NOS	8912 (537)	4394 (60.3)	3562 (52.2)	524 (42.1)	373 (36.2)	59 (26.8)	
Signet ring cell adenocarcinoma	4387 (26.4)	1619 (22.2)	2059 (30.2)	305 (24.5)	318 (30.8)	86 (39.1))	
Linitis plastica	1113 (6.7)	83 (1.1)	91 (1.3)	25 (2)	16 (1.6)	5 (2.3)	
Adenocarcinoma, intestinal type	751 (4.5)	424 (5.8)	372 (5.5)	150 (12.1)	140 (13.6)	27 (12.3)	
Adenocarcinoma, diffuse type	220 (1.3)	251 (3.4)	296 (4.3)	92 (7.4)	88 (8.5)	24 (10.9)	
Other	1213 (7.3)	511 (7)	439 (6.4)	148 (11.9)	96 (9.3)	19 (8.6)	
**T stage, 8th ed**							< **0.0001**
Tx	6736 (40.6)	3637 (49.9)	3036 (44.5)	28 (2.3)	32 (3.1)	3 (1.4)	
T1-2	3195 (19.3)	1513 (20.8)	1512 (22.2)	81 (6.5)	66 (6.4)	23 (10.4)	
T3-4	6665 (40.1)	2132 (29.3)	2271 (33.3)	1135 (91.2)	933 (90.5)	194 (88.2)	
**N stage, 8th ed**							< **0.0001**
N0	5744 (34.6)	2870 (39.4)	2519 (36.9)	180 (14.5)	139 (13.5)	36 (16.4)	
N1-2	5211 (31.4)	1843 (25.3)	2397 (35.2)	473 (38)	390 (37.8)	108 (49.1)	
N3	1293 (7.8)	69 (0.9)	136 (2)	539 (43.3)	474 (46)	75 (34.1)	
Nx	4348 (26.2)	2500 (34.3)	1767 (25.9)	52 (4.2)	28 (2.7)	1 (0.5)	
**Grade**							< **0.0001**
Well/moderate differentiated	2571 (15.5)	1171 (16.1)	963 (14.1)	249 (20)	164 (15.9)	24 (10.9)	
Poorly/undifferentiated	9925 (59.8)	4944 (53.5)	4109 (60.3)	924 (74.5)	816 (79.2)	180 (81.9)	
Unknown	4100 (24.7)	2217 (30.4)	1747 (25.6)	69 (5.5)	51 (4.9)	16 (7.2)	
**Metastatic spread**							< **0.0001**
Distant lymphnodes	1478 (8.9)	511 (7)	625 (9.2)	145 (11.7)	158 (15.3)	39 (17.7)	
Distant metastases	12,421 (74.8)	5605 (77)	4868 (71.4)	1002 (80.5)	787 (76.3)	159 (72.3)	
Distant lymphnodes + metastases	2697 (16.3)	1166 (16)	1326 (49.2)	97 (3.6)	86 (3.2)	22 (10)	
**Type of gastrectomy**							< **0.0001**
Partial gastrectomy				951 (76.4)	760 (73.7)	129 (58.6)	
Total (or near-total) gastrectomy				293 (23.6)	271 (26.3)	91 (41.4)	
**Number of retrieved lymphnodes**							< **0.0001**
≤ 15				801 (64.4)	590 (57.2)	111 (50.5)	
> 15				443 (35.6)	441 (42.8)	109 (49.5)	
**Radical intent**							< **0.0001**
No				620 (49.8)	426 (41.3)	81 (36.8)	
Yes				624 (50.2)	605 (58.7)	139 (63.2)	
Response to NAT*							
Regression						48 (21.8)	
Stable						103 (46.8)	
Progression						69 (31.4)	
**Response to chemotherapy**							
No			4260 (62.5)				
Yes			2559 (37.5)				

Statistically significant *p* values are given in bold

*NOS* Not otherwise specified.

*NAT: Neo-adjuvant chemotherapy.

### Treatment groups

A total of 9314 (56.1%) patients underwent CHT, surgery, radiotherapy (RT), or a combined treatment (eTable 1). CHT was performed in 8070 patients (51.4%) and as single treatment in 5946 patients (35.8%). 2495 (15%) patients underwent surgery, with a partial gastrectomy performed in most patients (n = 1840/2495; 73.75%). An associated lymphadenectomy with more than 16 retrieved lymph-nodes was achieved in 993 patients (39.8%; Table 1).

The surgical category was further divided into three subgroups: surgery alone, PG and SG. In addition to surgery alone (n = 1244), 1251 patients underwent a combined treatment with a PG or SG performed in 1031 (6.2%) and in 220 patients (1.3%), respectively. When radiotherapy was carried out, it was part of a multimodal treatment in most patients (n = 1149/1679, 68.4%).

A complicated disease and a worse performance status were shown, as expected, in patients who only underwent supportive care (BSC group: 72.4% and 51%, respectively; *p* < 0.0001; Table 1). Similar tumor characteristics were observed in the three surgical groups, but the gastrectomy alone group had a significant rate of patients with a more advanced age than those undergoing PG and SG (> 65yo: 66.8%, 38.9%, 30.9%, respectively; *p* < 0.0001) and poor PS (48.6%, 22.7%, 15.9%, respectively; *p* < 0.0001).

In the SG group, CHT administration was allowed to obtain a stable disease in 46.8% and a disease regression in 21.8% of patients (Table 1). In this group, more patients underwent a total gastrectomy than the other two groups (41.4% in SG vs 23.6% and 26.3% in the gastrectomy alone and PG groups, respectively; *p* < 0.0001), and a radical surgical intent was pursued in the majority of patients (63.2%).

### Survival outcomes in the global population

**Figure 1:**
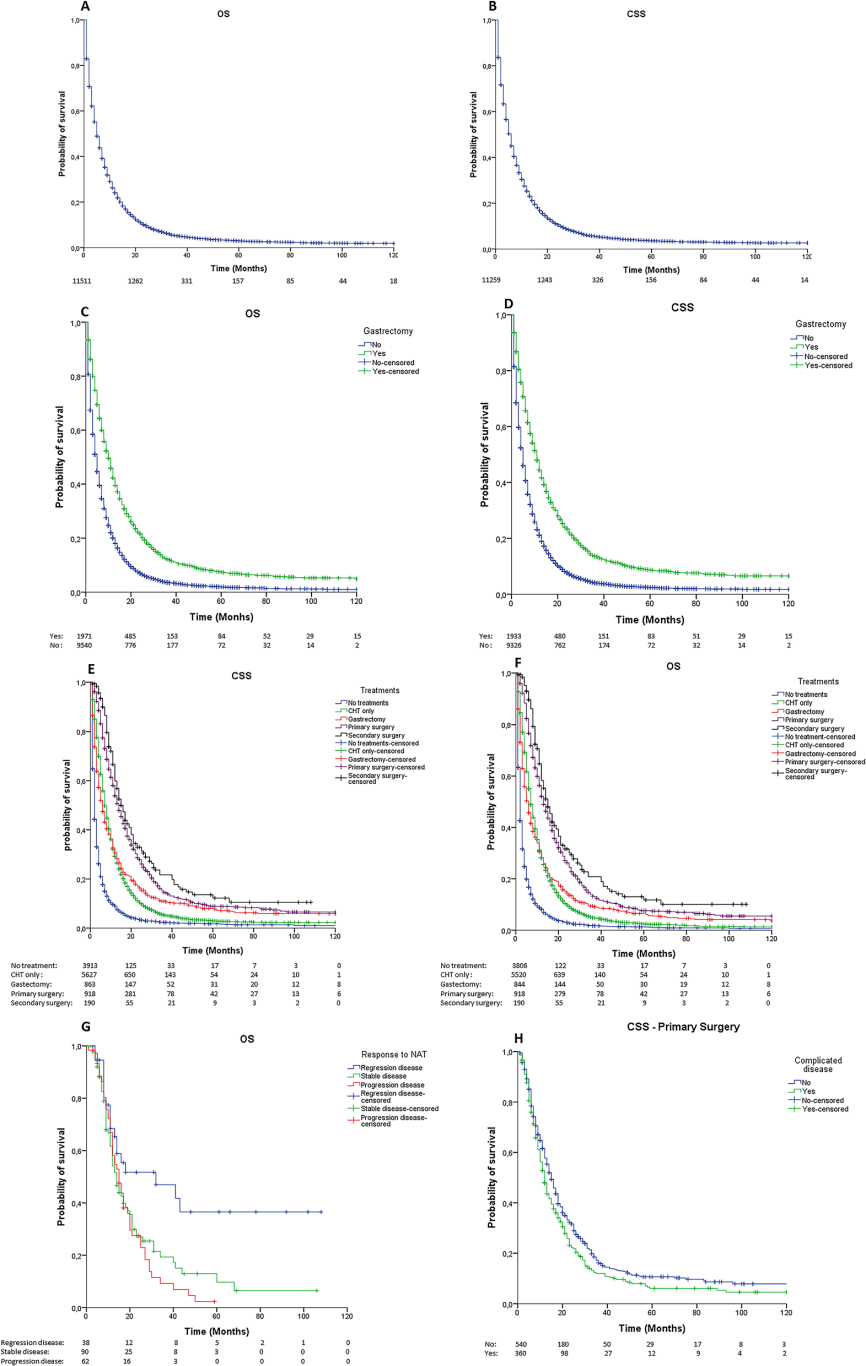
**A**, **B** Kaplan-Meier curve of overall survival (A) and cancer-specific survival (B). **C**, **D** Kaplan-Meier curves comparing OS (A) and CSS (B) between patients underwent or not gastrectomy (log-rank *p* < 0.0001). **E**, **F** Kaplan-Meier curves of OS (A) and CSS (B) among the different treatment groups (log-rank *p* < 0.0001). **G** Kaplan-Meier curves of OS comparing different pre-operative chemotherapy results in patients underwent secondary gastrectomy (log-rank *p* = 0.007). **H** Kaplan-Meier curves of OS comparing the effect caused by a complicated disease among patients underwent primary gastrectomy (log-rank *p* = 0.009)

After a median follow-up of 5 months (1–142 months), the median overall survival (OS, n = 11,511) was 5 months (IC95% = 4.8–5.2; Fig. 1A), and the median cancer-specific survival (CSS, n = 11,259) was 6 months (IC95% = 5.8–6.2; Fig. 1B). The overall and cancer-related mortality rates were 89.7% and 84.3%, respectively. Patients in the last period of the study (2011–2015) with good PS and without a complicated disease showed a significantly better OS and CSS (*p* < 0.001; Table 2).

**Table 2:** Kaplan–Meier estimates: median overall survival and median cancer-specific survival compared in different subgroups.

	Median OS, months (IC 95%)	*p*	Median CSS, months (IC 95%)	*p*
**Year of diagnosis**		< **0.001**		< **0.001**
2004–2006	5 (4.7–5.3)		5 (4.7–5.3)	
2007–2010	5 (4.7–5.3)		6 (5.7–6.3)	
2011–2015	6 (5.7–6.3)		6 (5.7–6.3)	
**Best supportive care (BSC)**		< **0.001**		< **0.001**
Yes	2 (1.9–2)		2 (1.9–2)	
No	8 (7.7–8.2)		8 (7.7–8.2)	
**Surgery**		< **0.001**		< **0.001**
Yes	10 (9.4–10.7)		11 (10.3–11.7)	
No	5 (4.9–5.1)		5 (4.8–5.2)	
**Chemotherapy (CHT)**		** < 0.001**		< **0.001**
Yes	8 (7.8–8.2)		8 (7.7–8.3)	
No	2 (1.9–2.1)		3 (2.9–3.1)	
**Radiotherapy (RT)**		< **0.001**		**0.002**
Yes	6 (5.5–6.5)		6 (5.5–6.5)	
No	5 (4.8–5.2)		6 (5.8–6.2)	
**Timing gastrectomy/chemotherapy**		< **0.001**		< **0.001**
BSC	2 (1.9–2.1)		2 (1.9–2.1)	
CHT + / − RT	7 (6.8–7.2)		7 (6.8–7.2)	
Gastrectomy	6 (5.3–6.7)		6 (5.3–6.7)	
PG	13 (11.9–14.0)		14 (12.9–15.1)	
SG	15 (12.7–17.3)		16 (13.6–18.4)	
**Type of gastrectomy**		0.453		0.549
Total (or near total)	10 (8.9–11.1)		11 (9.9–10.1)	
Partial	10 (9.2–10.8)		11 (10.1–11.9)	
**Number of retrieved lymphnodes**		< **0**.**001**		< **0**.**001**
≤ 15	9 (8.1–9.9)		10 (9–11)	
> 15	12 (11–13)		12 (10.8–13.2)	
**Radical intent**		< **0**.**001**		< **0**.**001**
No	9 (7.9–10)		10 (8.9–11-1)	
Yes	11 (10.1–11.8)		11 (10.1–11.9)	
**Performance status**		< **0.001**		< **0.001**
Good	6 (5.8–6.2)		7 (6.7–7.2)	
Poor	3 (2.8–3-1)		4 (3.8–6.2)	
**Complicated disease**		< **0.001**		< **0.001**
No	7 (6.6–7.3)		7 (6.6–7.3)	
Yes	5 (4.8–5-2)		5 (4.8–5.2)	
**Response to NAT**		**0.007**		**0.006**
Stable disease	14 (11.5–16.4)		14 (11–17)	
Disease regression	32 (1–65.2)		32 (1–64.4)	
Disease progression	15 (11.3–18.6)		15 (12.4–17.5)	

Statistically significant *p* values are given in bold

### Survival by treatment groups

Gastrectomy was the single treatment with the best outcomes for OS (median 10 months; IC95% = 9.4–10.7; *p* < 0.001; Table 2, Fig. 1C) and CSS (median 11 months; IC95% = 10.3–11.7, *p* < 0.001; Table 2, Fig. 1D), regardless if a partial or total gastrectomy was performed (OS *p* = 0.453; CSS *p* = 0.549; Table 2). When considering the combined treatments, OS was significantly higher in the SG (median 15 months; IC95% = 12.7–17.3; *p* < 0.001; Table 2, Fig. 1E) than in the PG (median 13 months; IC95% = 11.9–14), Chemo + /-radiotherapy (median 7 months; IC95% = 6.8–7.2) and gastrectomy alone (median 6 months; IC95% = 5.3–6.7) groups, respectively. The CSS rates showed a similar trend (Table 2, Fig. 1F).

Figure 1G shows the stratifications of patients who underwent SG based on the CHT effect (*p* = 0.007), which clearly favors patients who experienced disease regression before surgery, while Fig. 1H shows the significant effect (*p* = 0.006) induced by the complicated disease variable in patients who underwent PG.

### Univariate and multivariate analysis (overall population)

The univariate analysis on the Cox regression model showed that SG was related to better OS (HR = 0.22, IC95% = 0.18–0.26, *p* < 0.001; Table 3) and CSS (HR = 0.22, IC95% = 0.19–0.26, *p* < 0.001; Table 3). Other prognostic factors were found, such as type of metastatic spread, site of tumor, histology, grading, T and N stage, year of diagnosis, age, race, marital and insurance status, PS, and complicated disease (Table 3). After adjusting other variables in the multivariate Cox analysis, SG still significantly improved for both OS (HR = 0.22, IC95% = 0.18–0.26, *p* < 0.001; Table 3) and CSS (HR = 0.22, IC95% = 0.18–0.27, *p* < 0.001; Table 3).

**Table 3:** Prognostic factors for overall and cancer specific survival in the overall study population.

Variable	Overall survival				Cancer-specific survival			
	Univariate		Multivariate *n = 8365		Univariate		Multivariate* n = 8182	
	HR (95% CI)	*p*	HR (95% CI)	*p*	HR (95% CI)	*p*	HR (95% CI)	*p*
**Year of diagnosis**								
2004–2006	Reference		Reference		Reference		Reference	
2007–2010	0.91 (0.87–0.96)	< **0.001**	0.93 (0.88–0.98)	**0.012**	0.90 (0.86–0.95)	< **0.001**	0.91 (0.86–0.97)	**0.002**
2011–2015	0.87 (0.83–0.91)	< **0.001**	0.90 (0.85–0.96)	**0.001**	0.86 (0.81–0.90)	< **0.001**	0.88 (0.83–0.93)	< **0.001**
**Sex**								
Male	Reference				Reference			
Female	0.99 (0.95–1.03)	0.678			0.99 (0.95–1.03)	0.691		
**Age**								
< 65	Reference				Reference			
≥ 65	1.26 (1.21–1.31)	< **0.001**			1.23 (1.18–1.28)	< **0.001**		
**Race**								
White	Reference		Reference		Reference			
Black	0.98 (0.93–1.04)	0.509	1.03 (0.97–1.1)	0.293	0.96 (0.91–1.02)	0.147		
Other	0.92 (0.88–0.97)	**0.002**	0.94 (0.88–0.99)	**0.042**	0.93 (0.88–0.98)	**0.005**		
**Marital status**								
Unmarried	Reference				Reference			
Married	0.88 (0.84–0.92)	< **0.001**			0.88 (0.85–0.92)	< **0.001**		
**Insurance status**								
Insured	Reference				Reference			
Uninsured	1.10 (1.05–1.15)	< **0.001**			1.12 (1.07–1.17)	< **0.001**		
**Performance status**								
Good	Reference		Reference		Reference		Reference	
Poor	1.38 (1.33–1.44)	< **0.001**	1.19 (1.13–1.26)	< **0.001**	1.36 (1.3–1.42)	< **0.001**	1.16 (1.1–1.22)	< **0.001**
**Complicated disease**								
No	Reference		Reference		Reference		Reference	
Yes	1.3 (1.25–1-36)	< **0.001**	1.01 (1.04–1.15)	< **0.001**	1.32 (1.26–1.37)	< **0.001**	1.1 (1.04–1.16)	< **0.001**
**Site of tumor**								
Fundus-body	Reference				Reference			
Antrum-pylorus	0.95 (0.90–1.01)	0.107			0.96 (0.90–1.02)	0.145		
Overlapping lesion of the stomach	1.10 (1.03–1.17)	**0.008**			1.10 (1.03–1.18)	**0.008**		
Stomach, NOS	1.10 (1.04–1.16)	**0.010**			1.10 (1.04–1.16)	**0.001**		
**Histology**								
Adenocarcinoma/Carcinoma, NOS	Reference		Reference		Reference		Reference	
Signet ring cell adenocarcinoma	0.97 (0.93–1.01)	0.172	1.02 (0.96–1.08)	0.51	0.98 (0.93–1.02)	0.329	1.02 (0.96–1.08)	0.6
Linitis plastica	1.16 (0.99–1.36)	0.076	1.20 0.99–1.46)	0.062	1.13 (0.96–1.34)	0.149	1.16 (0.95–1.42)	0.147
Adenocarcinoma, intestinal type	0.78 (0.72–0.84)	< **0.001**	0.90 (0.82–0.98)	**0.021**	0.77 (0.71–0.83)	< **0.001**	0.89 (0.81–0.97)	**0.011**
Adenocarcinoma, diffuse type	0.83 (0.76–0.91)	< **0.001**	0.95 (0.85–1.05)	0.313	0.83 (0.75–0.91)	< **0.001**	0.93 (0.83–1.04)	0.21
Other	0.86 (0.80–0.93)	< **0.001**	0.9 (0.82–0.99)	**0.034**	0.87 (0.80–0.94)	**0.001**	0.91 (0.83–1.00)	0.054
**T stage, 8th ed**								
Tx	Reference				Reference			
T1-2	0.83 (0.78–0.87)	< **0.001**			0.82 (0.78–0.87)	< **0.001**		
T3-4	0.77 (0.74–0.81)	< **0.001**			0.77 (0.74–0.81)	< **0.001**		
**N stage, 8th ed**								
N0	Reference		Reference		Reference		Reference	
N1-2	0.87 (0.83–0.91)	< **0.001**	0.98 (0.93–1.04)	0.536	0.87 (0.82–0.91)	< **0.001**	0.98 (0.92–1.04)	0.468
N3	0.73 (0.68–0.78)	< **0.001**	1.21 (1.1–1.33)	< **0.001**	0.73 (0.68–0.78)	< **0.001**	1.22 (1.11–1.35)	< **0.001**
Nx	1.23 (1.16–1.29)	< **0.001**	1.07 (1–1.14)	**0.039**	1.24 (1.18–1.30)	< **0.001**	1.06 (0.99–1.14)	0.071
**Grade**								
Well/Moderate differentiated	Reference		Reference		Reference	< **0.001**	Reference	
Poorly/Undifferentiated	1.17 (1.10–1.23)	< **0.001**	1.30 (1.22–1.38)	< **0.001**	1.18 (1.12–1.25)		1.32 (1.24–1.40)	< **0.001**
**Metastatic spread**								
Distant lymphnodes	Reference		Reference		Reference		Reference	
Distant metastases	1.37 (1.28–1.47)	< **0.001**	1.19 (1.01–1.29)	< **0.001**	1.41 (1.31–1.52)	< **0.001**	1.22 (1.12–1.33)	< **0.001**
Distant lymphnodes + metastases	1.50 (1.38–1.62)	< **0.001**	1.36 (1.24–1.50)	< **0.001**	1.55 (1.42–1.68)	< **0.001**	1.41 (1.28–1.56)	< **0.001**
**Treatment**								
BSC	Reference		Reference		Reference		Reference	
CHT + /- RT	0.43 (0.42–0.45)	< **0.001**	0.42 (0.4–0.44)	< **0.001**	0.44 (0.42–0.46)	< **0.001**	0.42 (0.40–0.45)	< **0.001**
Gastrectomy	0.43 (0.40–0.47)	< **0.001**	0.40 (0.36–0.44)	< **0.001**	0.43 (0.39–0.46)	< **0.001**	0.39 (0.35–0.43)	< **0.001**
Primary gastrectomy	0.27 (0.25–0.29)	< **0.001**	0.25 (0.23–0.28)	< **0.001**	0.27 (0.25–0.29)	< **0.001**	0.25 (0.22–0.28)	< **0.001**
Secondary gastrectomy	0.22 (0.18–0.26)	< **0.001**	0.22 (0.18–0.26)	< **0.001**	0.22 (0.19–0.26)	< **0.001**	0.22 (0.18–0.27)	< **0.001**

Statistically significant *p* values are given in bold

*NOS* Not otherwise specified, *BSC* Best supportive care, *CHT* Chemotherapy, *RT* Radiotherapy.

*Forward selection model.

Year of diagnosis, race, PS, complicated disease, histology, grading, N stage, and metastatic spread were confirmed as significant prognostic factors in the multivariate model.

### Univariate e multivariate analysis (surgery specific)

A specific analysis involving only the three surgical groups is reported in Table 4. The univariate analysis showed SG related to better OS (HR = 0.49, IC95% = 0.41–0.59, *p* < 0.001; Table 4) and CSS (HR = 0.5, IC95% = 0.42–0.61, *p* < 0.001; Table 4). Other prognostic factors in this analysis were year of diagnosis, age, insurance status, PS, complicated disease, tumor site, histology, T stage, N stage, grade, type of metastatic spread, number of retrieved lymph-nodes, and radical intent. The type of gastrectomy performed (partial or total) was not related to survival effect (OS *p* = 0.46, CSS *p* = 0.61). In the multivariate analysis, SG still improved both OS (HR = 0.5, IC95% = 0.41–0.61, *p* < 0.001; Table 4) and CSS (HR = 0.53, IC95% = 0.43–0.65, *p* < 0.001; Table 4). The analysis also showed the following characteristics were unfavorably related to survival for both OS and CSS: complicated disease, overlapping lesions, N3 stage, undifferentiated grade, distant metastatic spread, and a limited lymphadenectomy.

**Table 4:** Prognostic factors for overall and cancer specific survival in patients underwent surgery for stage IV gastric cancer.

Variable	Overall survival				Cancer-specific survival			
	Univariate		Multivariariate* n = 1839		Univariate		Multivariate* n = 1805	
	HR (95% CI)	*p*	HR (95% CI)	*p*	HR (95% CI)	*p*	HR (95% CI)	*p*
**Year of diagnosis**								
2004–2006	Reference				Reference		Reference	
2007–2010	0.88 (0.79–0.98)	**0.026**			0.86 (0.77–0.96)	**0.010**	0.95 (0.84–1.07)	0.366
2011–2015	0.77 (0.68–0.87)	< **0.001**			0.73 (0.64–0.83)	< **0.001**	0.82 (0.71–0.94)	**0.004**
**Sex**								
Male	Reference				Reference			
Female	1.03 (0.94–1.13)	0.512			1.05 (0.95–1.16)	0.308		
**Age**								
< 65	Reference		Reference		Reference			
≥ 65	1.26 (1.14–1.38)	< **0.001**	1.15 (1.03–1.27)	**0.01**	1.21 (1.09–1.33)	< **0.001**		
**Race**								
White	Reference				Reference			
Black	0.91 (0.8–1.04)	0.175			0.87 (0.75–1)	0.053		
Other	0.89 (0.79–1)	0.064			0.9 (0.8–1.02)	0.092		
**Marital status**								
Unmarried	Reference				Reference			
Married	0.94 (0.85–1.04)	0.21			0.95 (0.86–1.06)	0.951		
**Insurance status**								
Insured	Reference				Reference			
Uninsured	1.14 (1.03–1.25)	**0.009**			1.18 (1.07–1.30)	**0.001**		
**Performance status**								
Good	Reference	< **0.001**			Reference			
Poor	1.35 (1.24–1.5)				1.32 (1.19–1.47)	< **0.001**		
**Complicated disease**								
No	Reference		Reference		Reference		Reference	
Yes	1.25 (1.14–1.38)	< **0.001**	1.14 (1.03–1.27)	**0.014**	1.27 (1.15–1.4)	< **0.001**	1.14 (102–1.27)	**0.022**
**Site of tumor**								
Fundus-Body	Reference		Reference		Reference		Reference	
Antrum-Pylorus	1.13 (0.97–1.31)	0.111	1.13 (0.97–1.32)	0.1	1.12 (0.97–1.3)	0.163	1.13 (0.96–1.32)	0.143
Overlapping lesion of the stomach	1.40 (1.18–1.68)	< **0.001**	1.32 (1.1–1.59)	**0.003**	1.4 (1.17–1.67)	< **0.001**	1.3 (1.08–1.57)	**0.005**
Stomach, NOS	1.21 (1.04–1.41)	**0.012**	1.16 (0.99–1.35)	0.073	1.2 (1.02–1.4)	**0.024**	1.14 (0.97–1.35)	0.114
**Histology**								
Adenocarcinoma/Carcinoma, NOS	Reference				Reference			
Signet ring cell adenocarcinoma	1.17 (1.04–1.32)	**0.008**			1.19 (1.05–1.34)	**0.006**		
Linitis plastica	1.53 (1.09–2.12)	**0.013**			1.33 (0.98–1.99)	0.067		
Adenocarcinoma, intestinal type	0.91 (0.78–1.06)	0.233			0.89 (0.75–1.05)	0.158		
Adenocarcinoma, diffuse type	0.98 (0.81–1.18)	0.818			1.01 (0.84–1.23)	0.883		
Other	0.88 (0.74–1.05)	0.158			0.89 (0.74–1.06)	0.200		
**T stage, 8th ed**								
Tx	Reference				Reference			
T1-2	0.60 (0.41–0.87)	**0.007**			0.57 (0.39–0.84)	**0.005**		
T3-4	1.12 (0.82–1.54)	0.464			1.14 (0.82–1.58)	0.435		
**N stage, 8th ed**								
N0	Reference		Reference		Reference		Reference	
N1-2	1.23 (1.05–1.43)	**0.009**	1.23 (1.04–1.45)	**0.016**	1.23 (1.05–1.44)	**0.012**	1.21 (1.02–1.45)	**0.03**
N3	1.56 (1.33–1.81)	< **0.001**	1.72 (1.44–2.05)	< **0.001**	1.58 (1.35–1.85)	< **0.001**	1.7 (1.42–2.05)	< **0.001**
Nx	1.79 (1.33–2.42)	< **0.001**	1.52 (1.08–2.15)	**0.018**	1.88 (1.38–2.55)	< **0.001**	1.55 (1.08–2.21)	**0.017**
**Grade**								
Well/Moderate differentiated	Reference		Reference		Reference	< **0.001**	Reference	
Poorly/Undifferentiated	1.36 (1.2–1.55)	< **0.001**	1.39 (1.21–1.59)	< **0.001**	1.41 (1.23–1.61)		1.38 (1.2–1.58)	< **0.001**
**Metastatic spread**								
Distant lymphnodes	Reference		Reference		Reference		Reference	
Distant metastases	1.41 (1.22–1.62)	< **0.001**	1.38 (1.19–1.6)	< **0.001**	1.44 (1.24–1.67)	< **0.001**	1.48 (1.24–1.69)	< **0.001**
Distant lymphnodes + metastases	1.63 (1.32–2.01)	< **0.001**	1.55 (1.28–1.93)	< **0.001**	1.64 (1.32–2.04)	< **0.001**	1.54 (1.23–1.94)	< **0.001**
**Type of gastrectomy**								
Partial	Reference				Reference			
Total (near total)	1.04 (0.94–1.16)	0.468			1.03 (0.92–1.15)	0.615		
**Number of retrieved lymphnodes**								
≤ 15	Reference		Reference		Reference		Reference	
> 15	0.82 (0.75–0.9)	< **0.001**	0.70 (0.63–0.79)	< **0.001**	0.83 (0.75–0.92)	< **0.001**	0.71 (0.64–0.80)	< **0.001**
**Radical intent**								
No	Reference				Reference			
Yes	0.86 (0.78–0.94)	**0.002**			0.87 (0.78–0.96)	**0.005**		
**Type of surgery**								
Gastrectomy alone	Reference		Reference		Reference		Reference	
Primary gastrectomy	0.6 (0.55–0.67)	< **0.001**	0.6 (0.54–0.66)	< **0.001**	0.61 (0.55–0.68)	< **0.001**	0.59 (0.52–0.65)	< **0.001**
Secondary gastrectomy	0.49 (0.41–0.59)	< **0.001**	0.5 (0.41–0.61)	< **0.001**	0.5 (0.42–0. 61)	< **0.001**	0.53 (0.43–0.65)	< **0.001**

Statistically significant *p* values are given in bold

*NOS* Not otherwise specified.

*Forward selection model.

In Table 5 is reported the univariate and multivariate analysis in the subgroup of responder patients. Prognostic factors in this univariate analysis were year of diagnosis, age, insurance status, complicated disease, histology, grade, type of metastatic spread and the type of treatment (SG). In the multivariate analysis, SG still improved both OS and CSS.

**Table 5:** Prognostic factors for overall and cancer specific survival in responder patients.

Variable	Overall survival				Cancer-specific Survival			
	Univariate		Multivariate* n = 1747		Univariate		Multivariate* n = 1747	
	HR (95% CI)	*p*	HR (95% CI)	*p*	HR (95% CI)	*p*	HR (95% CI)	*p*
**Year of diagnosis**								
2004–2006	Reference		Reference		Reference		Reference	
2007–2010	0.87 (0.77–0.99)	**0**.**028**	0.85 (0.74–0.98)	**0**.**029**	0.86 (0.76–0.98)	**0**.**020**	0.85 (0.74–0.98)	**0**.**026**
2011–2015	0.79 (0.70–0.90)	< **0**.**001**	0.81 (0.70–0.93)	**0**.**003**	0.79 (0.70–0.90)	< **0**.**001**	0.81 (0.70–0.93**)**	**0**.**003**
**Sex**								
Male	Reference				Reference			
Female	0.99 (0.91–1.09)	0.907			1.00 (0.92–1.01)	0.949		
**Age**								
< 65	Reference				Reference			
≥ 65	0.89 (0.81–0.98)	**0**.**016**			0.87 (0.79–0.96)	**0**.**005**		
**Race**								
White	Reference				Reference			
Black	0.93 (0.82–1.06)	0.274			0.91 (0.80–1.04)	0.165		
Other	1.03 (0.92–1.17)	0.591			1.05 (0.93–1.18)	0.475		
**Marital status**								
Unmarried	Reference				Reference			
Married	0.97 (0.88–1.07)	0.546			0.96 (0.88–1.07)	0.515		
**Insurance status**								
Insured	Reference				Reference			
Uninsured	1.13 (1.02–1.26)	**0**.**018**			1.14 (1.03–1.27)	**0**.**013**		
**Performance status**								
Good	Reference				Reference			
Poor	1.01 (0.91–1.13)	0.832			1.02 (0.91–1.13)	0.810		
**Complicated disease**								
No	Reference		Reference		Reference		Reference	
Yes	1.17 (1.07–1.28)	**0**.**001**	1.12 (1.01–1.25)	**0**.**036**	1.17 (1.07–1.28)	**0**.**001**	1.13 (1.01–1.26)	**0.032**
**Site of tumor**								
Fundus-Body	Reference				Reference			
Antrum-Pylorus	0.94 (0.82–1.07)	0.332			0.94 (0.82–1.07)	0.363		
Overlapping lesion of the stomach	1.10 (0.95–1.27)	0.218			1.10 (0.95–1.28)	0.249		
Stomach, NOS	1.05 (0.93–1.18)	0.449			1.05 (0.93–1.18)	0.468		
**Histology**								
Adenocarcinoma/Carcinoma, NOS	Reference		Reference		Reference		Reference	
Signet ring cell adenocarcinoma	1.18 (1.07–1.30)	**0**.**001**	1.22 (1.08–1.38)	**0**.**001**	1.17 (1.05–1.29)	**0**.**003**	1.21 (1.07–1.37)	**0**.**002**
Linitis plastica	1.43 (0.99–2.05)	0.055	1.18 (0.78–1.80)	0.428	1.46 (1.01–2.10)	**0**.**043**	1.20 (0.79–1.83)	0.384
Adenocarcinoma, intestinal type	0.79 §(0.65–0.96)	**0**.**020**	0.83 (0.67–1.04)	0.115	0.80 (0.65–0.97)	**0**.**025**	0.84 (0.67–1.04)	0.114
Adenocarcinoma, diffuse type	1.05 (0.86–1.28)	0.648	1.01 (0.81–1.26)	0.912	0.99 (0.80–1.21)	0.904	0.96 (0.76–1.21)	0.724
Other	1.01 (0.85–1.21)	0.876	1.02 (0.83–1.26)	0.866	1.01 (0.85–1.22**)**	0.885	1.01 (0.82–1.25)	0.906
**T stage, 8th ed**								
Tx	Reference				Reference			
T1-2	0.86 (0.77–0.97)	**0**.**013**			0.86 (0.76–0.96)	**0**.**010**		
T3-4	0.93 (0.84–1.03)	0.164			0.94 (0.85–1.04)	0.205		
**N stage, 8th ed**								
N0	Reference				Reference			
N1-2	0.98 (0.88–1.09)	0.721			0.98 (0.88–1.09)	0.745		
N3	0.94 (0.75–1.18)	0.611			0.95 (0.76–1.19)	0.665		
Nx	1.20 (1.07–1.35)	**0**.**002**			1.20 (1.07–1.36)	**0**.**002**		
**Grade**								
Well/Moderate differentiated	Reference		Reference		Reference		Reference	
Poorly/Undifferentiated	1.29 (1.13–1.47)	< **0**.**001**	1.18 (1.02–1.36)	**0**.**023**	1.28 (1.12–1.46)	< **0**.**001**	1.18 (1.02–1.36)	**0**.**026**
**Metastatic spread**								
Distant lymphnodes	Reference		Reference		Reference			
Distant metastases	1.16 (1.00–1.35)	**0**.**047**			1.20 (1.03–1.40)	**0**.**021**		
Distant lymphnodes + metastases	1.22 (1.03–1.46)	**0**.**025**			1.27 (1.06–1.52)	**0**.**009**		
**Treatment**								
Secondary gastrectomy	Reference		Reference		Reference		Reference	
CHT + / − RT	1.49 (1.23–1.81)	< **0**.**001**	1.46 (1.19–1.80)	< **0**.**001**	1.48 (1.22–1.80**)**	< **0**.**001**	1.44 (1.17–1.77)	**0**.**001**

Statistically significant *p* values are given in bold.

*Stepwise forward selection

### Propensity score matched analysis (PSM)

#### Primary Gastrectomy versus Secondary Gastrectomy

PSM was identified in 430 (n = 215 per group comparing PG vs SG, n = 189 per group valid for the survival analysis) matched patients at a 1:1 ratio out of a total of 1242 patients (eFigure 1, eTable 2). The L1 test measure was larger in the unmatched sample (0.794) than in the matched sample (0.749), indicating that the two groups were well balanced across all variables considered. The successful matching was confirmed during the analysis because there were no differences between the two groups regarding the patient’s characteristics (sex, age, race, PS, complicated disease), tumor characteristics (T, N, histology, grade, metastatic spread), and the type of surgery (type of gastrectomy, lymphadenectomy, radical intent).

**Figure 2:**
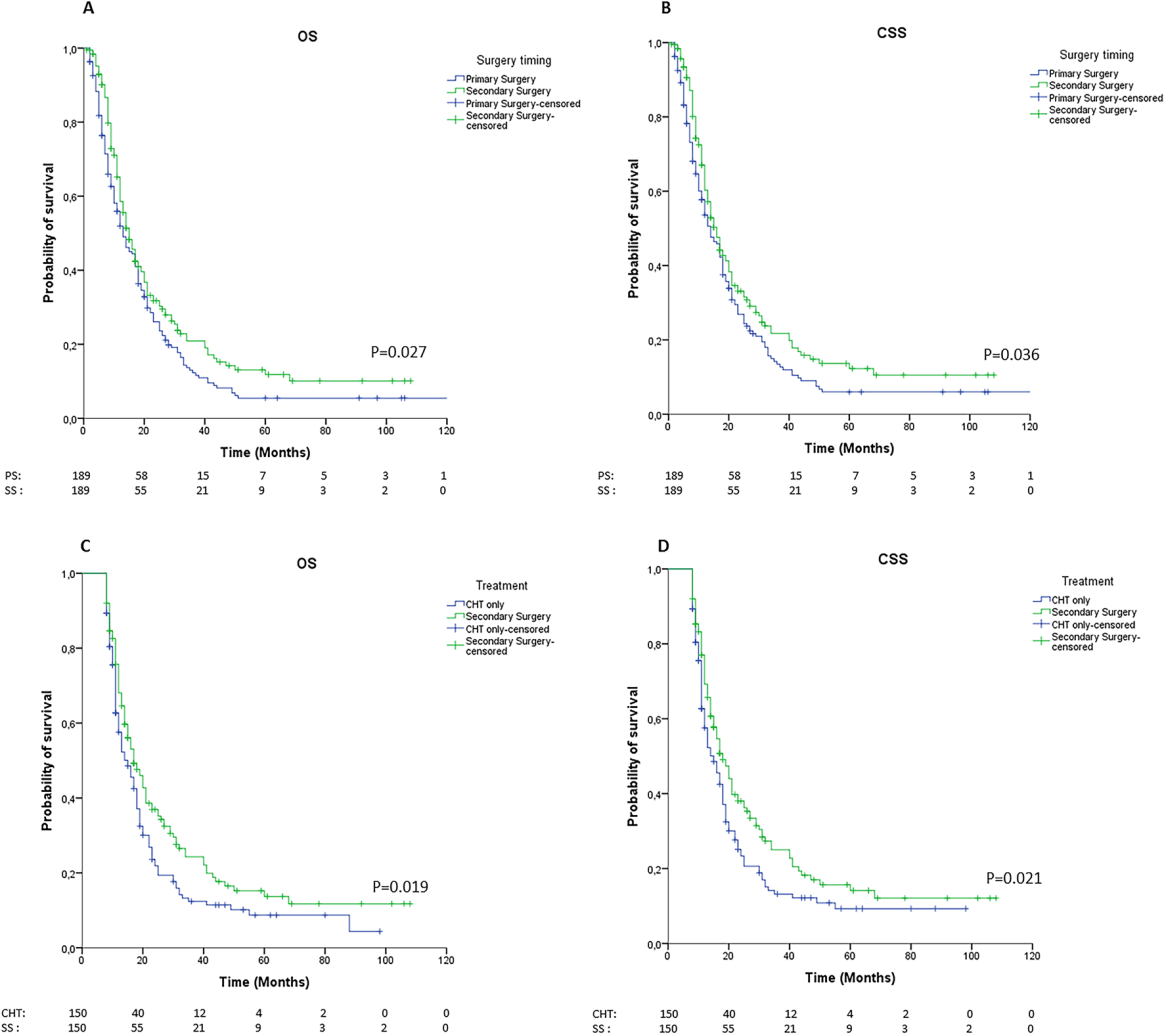
**A**, **B** Kaplan-Meier curves of OS (A, log-rank *p* = 0.027) and CSS (B, log-rank *p* = 0.036) comparing primary and secondary gastrectomy after PSM. **C**, **D** Kaplan-Meier curves of OS (A, log-rank *p* = 0.019) and CSS (B, log-rank *p* < 0.021) comparing secondary gastrectomy and chemotherapy after PSM

SG showed better OS (median 15 vs 13 months, *p* = 0.027; eTable 3, Fig. 2A) and CSS (median 16 vs 14 months, *p* = 0.036; eTable 3, Fig. 2B) than did PG. In the Cox analysis after PSM, SG was associated with significantly improved OS (HR = 0.78, IC95% = 0.62–0.98, *p* = 0.032) and CSS (HR = 0.79, IC95% = 0.62–0.99, *p* = 0.041).

#### Chemotherapy versus Secondary Gastrectomy

PSM was identified in 300 (n = 150 per group comparing CHT only vs SG) matched patients at a 1:1 ratio out of a total of 2299 patients (eFigure 2, eTable 4). The L1 test measure was larger in the unmatched sample (0.863) than in the matched sample (0.587), indicating that the two groups were well balanced across all variables considered.

The successful matching was confirmed during the analysis because there were no differences between the two groups regarding the patient’s characteristics (sex, age, race, PS) and tumor characteristics (histology, grade, metastatic spread).

SG showed better OS (median 17 vs 15 months, *p* = 0.019; eTable 5, Fig. 2C) and CSS (median 18 vs 15 months, *p* = 0.021; eTable 5, Fig. 2D) than did CHT. In the Cox analysis after PSM, SG was associated with significantly improved OS (HR = 0.74, IC95% = 0.58–0.96, *p* = 0.025) and CSS (HR = 0.74, IC95% = 0.57–0.97, *p* = 0.026).

## Discussion

The present study represents the largest sample (n = 16,596) to date to report outcomes for metastatic gastric cancer (mGC) and analyze its different treatment modalities. Patients who underwent gastrectomy obtained significant OS advantages over those who did not (median OS = 10 months; *p* < 0.001), and notably better results were achieved when combined with CHT. The multimodality approach proved an optimum strategy and can be further subdivided according to the timing of the surgical intervention: before (PG) or after (SG) CHT administration. Our PSM analysis showed a significant advantage in favor of SG (median OS = 15 months; *p* = 0.027).

### Role of gastrectomy

Most studies in the current literature feature comparisons of gastrectomy for mGC categorized into “nonresective” (NR) or “nonsurgical” (NS) groups, with the limitation of using mixed patient populations (BSC + /-CHT + /-other surgery). Nonetheless, some solid points that support our results have emerged, such as the finding that gastrectomy, overall, positively affects survival.

Chang^22^, in a Korean study involving 257 patients, reported that 165 patients who underwent gastrectomy had a longer median OS (12.7 months) than seen in the NR group (11.2 months; *p* = 0.01). Chiu^23^ showed a median OS in favor of the gastrectomy group (14.3 months, 95%CI = 8.0–20.7) versus an NS group (7.1 months, 95%CI = 6.2–8.0; *p* < 0.001). Yazici^24^ obtained an OS of 14 months (95%CI = 12.07–15.92) in the gastrectomy group versus 9 months (95%CI = 8.05–9.94; *p* < 0.001) in the NS group. Kulig^9^ reported an OS of 10.6 months (95%CI = 9.3–11.9) after gastrectomy versus 4.4 months (95%CI = 4.0–4.8; *p* < 0.001) in the NR group (HR = 2.923; 95%CI = 2.473–3.454).

It is difficult to understand the mechanisms by which gastrectomy may improve survival in this patient population, which is characterized by poor prognoses. Possible areas for future investigations include the following:

- A positive effect on symptom relief, including possible difficulties in oral feeding, obstruction, and anemia caused by the gastric tumoral mass,
- The prevention of sudden acute complications, and
- An overall reduction in the tumoral burden and its effect on the immune response of the organism and microbiota

### The multimodality approach

Interestingly, we found that patients undergoing CHT alone or gastrectomy alone have similar results in OS and CSS, as well as the same effect in the univariate and multivariate analyses. Then, we analyzed the combination of gastrectomy and CHT, observing a significant beneficial synergistic effect in our study.

Similarly, in a subgroup analysis, Hsu^25^ showed that patients treated with gastrectomy + CHT had a longer OS than those receiving gastrectomy or CHT alone or no treatments (*p* < 0.0001). The 1-year survival rate was 37.0% for the combined treatment group versus 2.9% for patients without any treatment. Chang^22^ stratified patients who underwent surgery according to CHT administration, achieving a survival increase of 8.6 months in the group including both treatments rather than BSC.

### Timing of surgery

Seo et al.^26^ reported data that seem consistent with our findings and that represent the only attempt to compare different treatment arms based on different multimodality and timing approaches. In their analysis, a better than 1-year survival rate was shown in SG patients in respect to the CHT group (*p* = 0.001), with a favorable trend even when compared with PG. To better evaluate combined approaches in this context, the Reductive Gastrectomy for Advanced Tumor in Three Asian Countries (REGATTA) trial^11, 27^ has been one of the most relevant research efforts. This randomized phase 3 trial at 44 centers in Japan, South Korea, and Singapore compared gastrectomy followed by CHT with CHT alone in patients with a single non-curable factor. The study started in February 2008 but was terminated in September 2013 after having failed to demonstrate survival benefits from the combined approach at the first interim analysis, which showed a median OS of 16.6 months (95%CI = 13.7–19.8) for patients assigned to CHT alone versus 14.3 months (95%CI = 11.8–16.3) for those assigned to gastrectomy plus CHT (HR = 1.09; 95%CI = 0.78–1.52; *p* = 0.70).

REGATTA^27^ was indisputably a landmark study, but instead of bringing clarity, it produced further debate while also demonstrating the difficulty of carrying out studies on this category of patients. The main limitation of the trial was its focus on a very restricted and select category of patients, which hindered generalization of its findings for application to common practice. However, attempts to increase patients’ survival rate have increasingly indicated that CHT plays a fundamental role, and that any other supporting procedure should avoid delaying the administration of chemotherapeutic protocols. Notably, patients who underwent gastrectomy as their primary treatment in the REGATTA trial had fewer CHT cycles, perhaps affecting their OS results.

Advances in CHT schemas have opened new therapeutic perspectives and strengthened the hypothesis that resection of the primary and/or metastatic lesions after successful CHT can improve survival.

Based on this evidence, Yoshida^12^ defined the concept of conversion surgery: “it is a surgical treatment aiming at an R0 resection after CHT for tumors that were originally unresectable or marginally resectable for technical and/or oncological reasons”.

Moreover, Yoshida^12^ proposed to divides stage IV GC in four categories based on different disease presentations: category 1 includes patients with metastatic but technically resectable disease; category 2 characterized by technically unresectable disease at the diagnosis suitable for induction CHT; category 3 includes patients with peritoneal dissemination while Category 4 patients with evidence of both peritoneal and other organ metastases.

In this setting, indications for conversion therapy might include patients from category 2 and selected patients from category 3 and category 4.

Yoshida^12^ highlights that conversion therapy, in eligible patients, might become the main treatment approach for stage IV GC. A prospective cohort study is currently ongoing in Asia (https://upload.umin.ac.jp/cgi-open-bin/ctr_e/ctr_view.cgi?recptno=R000005699), while it is desirable that similar initiatives will be started in the West, where stage IV GC is a common disease presentation.

In the current context, our research intended to clarify the possible expected survival outcomes using a multimodality approach that includes gastric resection in addition to planned CHT. When we evaluated gastrectomy as the only treatment provided, our analysis did not identify any benefit in comparison with salvage CHT alone. Instead, gastric resection combined with planned pre- or post-surgery CHT leads to better results in both OS and CSS compared to patients treated with CHT alone. The results of our study added new data to this scenario by identifying differences based on surgical timing in respect to CHT, with SG achieving statistically significant better results in the survival analysis, also through PSM, even when compared with PG. Moreover, most patients undergoing SG, in our analysis, pursued a radical intent after CHT. This group of patients showed the best results compared to all the other treatment strategies analyzed, especially after successful CHT.

Finally, we decided to analyze those patients presenting the best survival scenario and thus a PSM comparison, considering responder patients, was performed between SG and CHT alone groups. In this context, SG still showed significant advantages versus CHT alone, but the latter achieved optimal results with a mean difference in OS of two months only.

### Prognostic and influencing factors

The univariate and multivariate analyses in our study showed factors related to OS and CSS. In the overall study population, regardless of treatments strategies, the following patients and tumor characteristics were related to better survival (multivariate analysis): good PS, no evidence of a complicated disease, intestinal type, limited N stage, and limited metastatic spread. This analysis also showed the weight of different treatments. CHT alone and gastrectomy alone had the same effect, while their combination showed the best results.

When considering only those patients who underwent surgery, the following factors showed the best effects on OS and CSS: age < 65yo, no evidence of complicated disease, low N stage, differentiated tumor, and limited metastatic spread. Among the treatments, the determining prognostic factor was the administration of CHT. In our study, it was administered to almost half of the overall sample (48.6%), obtaining an average increase of survival of 5 more months than the BSC group and showing a synergistic action when combined with gastrectomy.

Conversely, gastrectomy alone, unless emergency procedures are needed, cannot be considered a correct approach to mGC patients, as no significant survival benefits can be expected (mean OS 6 months in gastrectomy alone vs 7 months in CHT alone), and it adds the risk of compromising the administration of systemic CHT.

The association between surgery and CHT showed the best results in patients of SG, as confirmed in our PSM. The most relevant factor in the SG was the preoperative CHT result, with excellent outcomes when a regression of the disease is achieved.

The analysis of surgery-related factors showed that type of gastrectomy (partial vs total) did not influence outcomes, while a procedure including extensive lymphadenectomy was associated to better OS and CSS.

In the literature, several published studies tried to identify eventual positive or negative prognostic factors. In two models resulting from an analysis of a series of patients undergoing CHT (Koo^28^, Lee^29^), the concomitant presence of the following factors was related to an estimated median survival of fewer than 3 months: ECOG performance status ≥ 2, high level of serum alkaline phosphatase, low level of serum albumin, lack of gastrectomy, presence of bone or lung metastasis, and ascites.

Moreover, authors of some studies reporting on surgery identified some possible characteristics that favorably affect OS and thus can be used as selection criteria for palliative gastrectomy. Hsu^25^ showed that in the G group, aged ≤ 58 years, preoperative albumin level > 3 g/dL, and use of CHT are favorable independent prognostic factors. Chiu^23^, evaluating the possible role of oncological biomarkers, reported improved survival rates in patients with normal preoperative values of CEA and/or CA19-9. Other studies evaluated the systemic inflammatory response indices: C-reactive protein (CRP), neutrophil-to-lymphocyte ratio (NLR), and the inflammation-based modified Glasgow Prognostic Score (mGPS: scoring system using CRP and albumin^30–33^. Notably, Baba^31^ identified a CRP cut-off value of 1.7 mg/dL as a short-term survival predictor, while Tanaka^32^ observed increased OS rates in patients with an NLR < 2.5.

Mimatsu^33^ reported a worse CSS in patients who underwent palliative gastrectomy, with an high mGPS in respect to patients with lower values.

Identifying subgroups of patients by number and location of metastases is more complex, and current studies have not found clear correlations allowing the generalization of results by these factors^34, 35^. The effects of these parameters thus cannot be rigorously predetermined; rather, they must be assessed patient by patient.

However, from a general point of view, an oncological patient’s performance status derives from two main components: the patient’s characteristics (age, comorbidities, etc.) and tumor details (the whole impact of the disease on the patient’ general conditions). It can thus be hypothesized that the greater the spread of the disease and the tumor burden, the greater the effect on the patient’s clinical status and thus the indirect effect on survival.

### Limitations and strengths

The present study is based on data collected from a population registry and thus from both direct and indirect variables mediated by a code system. The extrapolation of some derivatives for this study required combining information from multiple variables and deductive processes based on analysis methods already carried out in similar studies published in the literature.

If this system is characterized by extreme rigidity, it also allows a rigorous recording of patients’ data according to a well standardized manual-based system, and all information entered comes from institutions with personnel trained for this purpose.

This approach guarantees a high level of reliability and quality in the data collected. The major limitation is then the reduced availability of more detailed information on the characteristics of the patients and some specifics on the treatments carried out, the course of hospital stays, and complications.

In the two PSM analyses some factors were unbalanced. Particularly, the “year of diagnosis” in the comparison between PG vs SG groups could favor the SG group considering the developments in drugs and surgical techniques.

The T and N stages are relevant factors to be considered. However, they are difficult to be correctly evaluated in stage IV gastric cancer because in most patients they are only based on clinical features that often cannot be correctly assessed. As a result, caution must be taken when considering the T and N variables in the comparison between CHT and SG as well as in the prognostic factors evaluation from the overall population analysis.

Surgeons determine the indication of conversion surgery according to the depth of response, extent of metastatic disease, and performance status. Data available in the present study cannot allow us to accurately determine these factors, particularly as regards the degree of response to CHT. Consequently, the comparison between CHT and SG reported in our analysis suggests that tailored studies should now be conducted to better investigate this field.

Ultimately, the strength remains a remarkably high number of patients not otherwise analyzable by other studies, which lays the foundations for more refined targeted studies as RCTs.

## Conclusions

The surgical removal of the primary tumor shows a general positive effect on survival, which is however limited in patients undergoing gastrectomy alone and amplified when association with CHT is possible.

As shown in our study, a proportion of patients with mGC may benefit from adding gastrectomy to CHT. Accordingly, referral centers for GC with internal protocols approved by a multi-disciplinary team, after assessing the patient’s condition and determining that emergency surgery is not needed, can propose CHT first, followed by, in patients with at least a CHT-controlled disease, SG.

## Data availability 

The data that support the findings of this study are available from the corresponding author upon reasonable request.

## Supplementary Information

Supplementary information.
