# Supplementary material for: Gastrectomy for stage IV gastric cancer: a comparison of different treatment strategies from the SEER database
Source: Sci Rep. 2021 Mar 30;11:7150. doi: 10.1038/s41598-021-86352-6 (PMC8010081; doi:10.1038/s41598-021-86352-6)
Supplement: Supplementary file 1 — Supplementary information. [file 41598_2021_86352_MOESM1_ESM.docx]

*Online-Only Supplements*

**Gastrectomy for Stage IV Gastric Cancer: A Comparison of Different Treatment Strategies from the SEER Database**

Jacopo Desiderio^1,2*^, MD, Andrea Sagnotta^3,4*^, MD, PhD, Irene Terrenato^5^, MSc, Bruno Annibale^4^, MD, Stefano Trastulli^1^, MD, PhD, Federico Tozzi^6^, MD, PhD, Vito D’Andrea^2^, MD, Sergio Bracarda^7^, MD, Eleonora Garofoli^7^, MD, Yuman Fong^8^, MD, Yanghee Woo^8^, MD, Amilcare Parisi^1^, MD

*Jacopo Desiderio and Andrea Sagnotta have contributed equally to this work and should be considered co-ﬁrst authors.

^1^ St. Mary’s Hospital, Department of Digestive Surgery, Terni, Italy.

^2^ La Sapienza University of Rome, Department of Surgical Sciences – PhD program in advanced surgical technologies, Rome, Italy.

^3^ San Filippo Neri Hospital, Department of General Surgery and Surgical Oncology, Rome, Italy.
^4^ Department of Medical-Surgical Sciences and Translational Medicine, Sant'Andrea Hospital, Sapienza University of Rome, Rome, Italy.
^5^ Biostatistics and Bioinformatic Unit, Scientific Direction, IRCCS Regina Elena National Cancer Institute, Rome, Italy.

^6^ Department of Surgical Oncology and Endocrine Surgery, Mays Cancer Center - UT Health San Antonio, San Antonio, USA.

^7^ St. Mary’s Hospital, Department of Medical Oncology, Terni, Italy.
^8^City of Hope National Medical Center, Division of Surgical Oncology- Department of Surgery, Duarte- CA, USA.

| **Characteristics** | **Total**  **(n=16,596)** |
| --- | --- |
| **Best supportive care (BSC)**  Yes  No | 7,282 (43.9%)  9,314 (56.1%) |
| **Surgery**  No  Partial gastrectomy  Total (or near-total) gastrectomy | 14,101 (85%)  1,840 (11.1%)  655 (3.9%) |
| **Chemotherapy (CHT)**  Yes  No | 8,526 (48.6%)  8,070 (51.4%) |
| **Radiotherapy**  No  Yes | 14,917 (89.8%)  1,679 (10.2%) |
| **Timing Gastrectomy/Chemotherapy**  BSC  CHT +/- RT  Gastrectomy alone  Primary gastrectomy (PG)  Secondary gastrectomy (SG) | 7,282 (43.9)  6,819 (41.1%)  1,244 (7.5%)  1,031 (6.2%)  220 (1.3%) |

**eTable 1:** *Type of performed treatments*

| **Patient Characteristics after PSM (N=430)** | | | | | |  |
| --- | --- | --- | --- | --- | --- | --- |
|  | **Primary Gastrectomy**  **(N=215)** | | | **Secondary Gastrectomy**  **(N=215)** | |  |
|  | **N** | **%** | **N** | | **%** | **p** |
| **Year of diagnosis** |  |  |  | |  |  |
| 2004-2006 | 71 | 33.0 | 24 | | 11.2 | <0.001 |
| 2007-2010 | 78 | 36.3 | 65 | | 30.2 |  |
| 2011-2015 | 66 | 30.7 | 126 | | 58.6 |  |
| **Sex** |  |  |  | |  | 0.500 |
| Male | 110 | 51.2 | 103 | | 47.9 |  |
| Female | 105 | 48.8 | 112 | | 52.1 |  |
| **Age** |  |  |  | |  | 0.238 |
| <65 aa | 160 | 74.4 | 149 | | 69.3 |  |
| ≥65 aa | 55 | 25.6 | 66 | | 30.7 |  |
| **Race** |  |  |  | |  | 0.689 |
| White | 143 | 66.5 | 136 | | 63.3 |  |
| Black | 30 | 14.0 | 31 | | 14.4 |  |
| Other | 42 | 19.5 | 47 | | 22.3 |  |
| **Marital Status** |  |  |  | |  | 0.082 |
| Unmarried | 77 | 35.8 | 62 | | 28.8 |  |
| Married | 132 | 61.4 | 139 | | 64.7 |  |
| Unknown | 6 | 2.8 | 14 | | 6.5 |  |
| **Insurance Status** |  |  |  | |  | <0.001 |
| Insured | 130 | 60.5 | 180 | | 83.7 |  |
| Uninsured | 82 | 38.1 | 33 | | 15.3 |  |
| Unknown | 3 | 1.4 | 2 | | 0.9 |  |
| **Site of tumor** |  |  |  | |  | <0.001 |
| Fundus- Body | 38 | 17.7 | 54 | | 25.1 |  |
| Antrum-Pylorus | 85 | 39.5 | 52 | | 24.2 |  |
| Overlapping lesion of the stomach | 25 | 11.6 | 49 | | 22.8 |  |
| Stomach, NOS | 38 | 17.7 | 23 | | 10.7 |  |
| Other | 29 | 13.5 | 37 | | 17.2 |  |
| **Histology** |  |  |  | |  | 0.461 |
| Adenocarcinoma/Carcinoma, NOS | 66 | 30.7 | 57 | | 26.5 |  |
| Signet ring cell adenocarcinoma | 87 | 40.5 | 85 | | 39.5 |  |
| Linitis plastica | 2 | 0.9 | 5 | | 2.3 |  |
| Adenocarcinoma, intestinal type | 27 | 12.6 | 26 | | 12.1 |  |
| Adenocarcinoma, diffuse type | 14 | 6.5 | 24 | | 11.2 |  |
| Other | 19 | 8.8 | 18 | | 8.4 |  |
| **T stage, 8th ed** |  |  |  | |  | 0.486 |
| Tx | 5 | 2.3 | 3 | | 1.4 |  |
| T1-2 | 17 | 7.9 | 23 | | 10.7 |  |
| T3-4 | 193 | 89.3 | 189 | | 87.9 |  |
| **N stage, 8th ed** |  |  |  | |  | 0.144 |
| Nx | 6 | 2.8 | 1 | | 0.5 |  |
| N0 | 42 | 19.5 | 34 | | 15.8 |  |
| N1-2 | 92 | 42.8 | 106 | | 49.3 |  |
| N3 | 75 | 34.9 | 74 | | 34.4 |  |
| **Grade** |  |  |  | |  | 0.249 |
| Well/moderate differentiated | 33 | 15.3 | 23 | | 10.7 |  |
| Poorly/Undifferentiated | 171 | 79.5 | 176 | | 81.9 |  |
| Unknown | 11 | 5.1 | 16 | | 7.4 |  |
| **Metastatic spread** |  |  |  | |  | 0.442 |
| Distant lymphnodes | 35 | 16.2 | 37 | | 17.2 |  |
| Distant metastases | 165 | 76.7 | 156 | | 72.6 |  |
| Distant metastases + lymphnodes | 15 | 7.1 | 22 | | 10.2 |  |
| **Complicated disease** |  |  |  | |  | 0.160 |
| No | 144 | 67.0 | 130 | | 60.5 |  |
| Yes | 71 | 33.0 | 85 | | 39.5 |  |
| **Performance status** |  |  |  | |  | 0.894 |
| Good | 181 | 84.2 | 182 | | 84.7 |  |
| Poor | 34 | 15.8 | 33 | | 15.3 |  |
| **Type of gastrectomy** |  |  |  | |  | 0.001 |
| Total (or near total) | 55 | 74.4 | 88 | | 59.1 |  |
| Partial | 160 | 25.6 | 127 | | 40.9 |  |
| **Number of retrieved lymphnodes** |  |  |  | |  | 0.026 |
| ≤15 | 131 | 60.9 | 108 | | 50.2 |  |
| >15 | 84 | 39.1 | 107 | | 49.8 |  |
| **Radical intent** |  |  |  | |  | 0.095 |
| No | 96 | 44.7 | 79 | | 36.7 |  |
| Yes | 119 | 55.3 | 136 | | 63.3 |  |

**eTable 2:** *Patients characteristics after case-matched analysis comparing primary and secondary gastrectomy.*

|  | **Median OS, months**  **(CI 95%)** | ***p*** | **Median CSS, months**  **(CI 95%)** | ***p*** |
| --- | --- | --- | --- | --- |
|  |  |  |  |  |
| Primary Gastrectomy | 13 (10.3-15.7) | ***0.027*** | 14 (11.1-16.9) | ***0.036*** |
| Secondary Gastrectomy | 15 (12.7-17.3) |  | 16 (13.7-18.4) |  |

**eTable 3:** *Estimated Overall and Cancer-specific survival time in primary and secondary gastrectomy groups after PSM.*

| **Patient Characteristics after PSM (N=300)** | | | | | | |  |
| --- | --- | --- | --- | --- | --- | --- | --- |
|  | **Chemotherapy**  **(N=150)** | | **Secondary Gastrectomy**  **(N=150)** | |  | |  |
|  | **N** | **%** | **N** | **%** | | ***p*** | |
| **Year of diagnosis** |  |  |  |  | | 0.673 | |
| 2004-2006 | 22 | 14.7 | 18 | 12.0 | |  | |
| 2007-2010 | 49 | 22.7 | 46 | 30.7 | |  | |
| 2011-2015 | 79 | 52.7 | 86 | 57.3 | |  | |
| **Sex** |  |  |  |  | | 0.816 | |
| Male | 68 | 45.3 | 66 | 44.0 | |  | |
| Female | 82 | 54.7 | 84 | 56.0 | |  | |
| **Age** |  |  |  |  | | 1.000 | |
| <65 aa | 109 | 72.7 | 109 | 72.7 | |  | |
| ≥65 aa | 41 | 27.3 | 41 | 27.3 | |  | |
| **Race** |  |  |  |  | | 0.946 | |
| White | 86 | 57.3 | 88 | 58.7 | |  | |
| Black | 24 | 16.0 | 24 | 16.0 | |  | |
| Other | 38 | 25.3 | 37 | 24.7 | |  | |
| unknown | 2 | 1.3 | 1 | 0.7 | |  | |
| **Marital Status** |  |  |  |  | | 0.968 | |
| Unmarried | 43 | 28.7 | 45 | 30.0 | |  | |
| Married | 100 | 66.7 | 98 | 65.3 | |  | |
| Unknown | 7 | 4.7 | 7 | 4.7 | |  | |
| **Insurance Status** |  |  |  |  | | 0.631 | |
| Insured | 117 | 78.0 | 123 | 82.0 | |  | |
| Uninsured | 31 | 20.7 | 26 | 17.3 | |  | |
| Unknown | 2 | 1.3 | 1 | 0.7 | |  | |
| **Site of tumor** |  |  |  |  | | 0.448 | |
| Fundus- Body | 37 | 24.7 | 33 | 22.0 | |  | |
| Antrum-Pylorus | 45 | 30.0 | 39 | 26.0 | |  | |
| Overlapping lesion of the stomach | 25 | 16.7 | 36 | 24.0 | |  | |
| Stomach, NOS | 43 | 28.7 | 42 | 28.0 | |  | |
| **Histology** |  |  |  |  | | 0.089 | |
| Adenocarcinoma/Carcinoma, NOS | 62 | 41.3 | 43 | 28.7 | |  | |
| Signet ring cell adenocarcinoma | 57 | 38.0 | 56 | 37.3 | |  | |
| Linitis plastica | 3 | 2.0 | 3 | 2.0 | |  | |
| Adenocarcinoma, intestinal type | 9 | 6.0 | 18 | 12.0 | |  | |
| Adenocarcinoma, diffuse type | 13 | 8.7 | 17 | 11.3 | |  | |
| Other | 6 | 4.0 | 13 | 8.7 | |  | |
| **Grade** |  |  |  |  | | 0.280 | |
| Well/moderate differentiated | 13 | 8.7 | 16 | 10.7 | |  | |
| Poorly/Undifferentiated | 113 | 75.3 | 119 | 79.3 | |  | |
| Unknown | 24 | 16.0 | 15 | 10.0 | |  | |
| **Metastatic spread** |  |  |  |  | | 0.830 | |
| Distant lymphnodes | 29 | 19.3 | 25 | 16.7 | |  | |
| Distant metastases | 104 | 69.3 | 108 | 72.0 | |  | |
| Distant metastases + lymphnodes | 17 | 11.3 | 17 | 11.3 | |  | |
| **Performance status** |  |  |  |  | | 0.857 | |
| Good | 18 | 12.0 | 17 | 11.3 | |  | |
| Poor | 132 | 88.0 | 133 | 88.7 | |  | |

**eTable 4:** *Patients characteristics after case-matched analysis comparing chemotherapy and secondary gastrectomy.*

|  | **Median OS, months**  **(CI 95%)** | ***p*** | **Median CSS, months**  **(CI 95%)** | ***p*** |
| --- | --- | --- | --- | --- |
|  |  |  |  |  |
| Chemotherapy | 15 (12.2-17.8) | ***0.019*** | 15 (12.2-17.8) | ***0.021*** |
| Secondary Gastrectomy | 17 (13.9-20.1) |  | 18 (15.0-21.0) |  |

**eTable 5:** *Estimated Overall and Cancer-specific survival time in chemotherapy and secondary gastrectomy groups after PSM.*


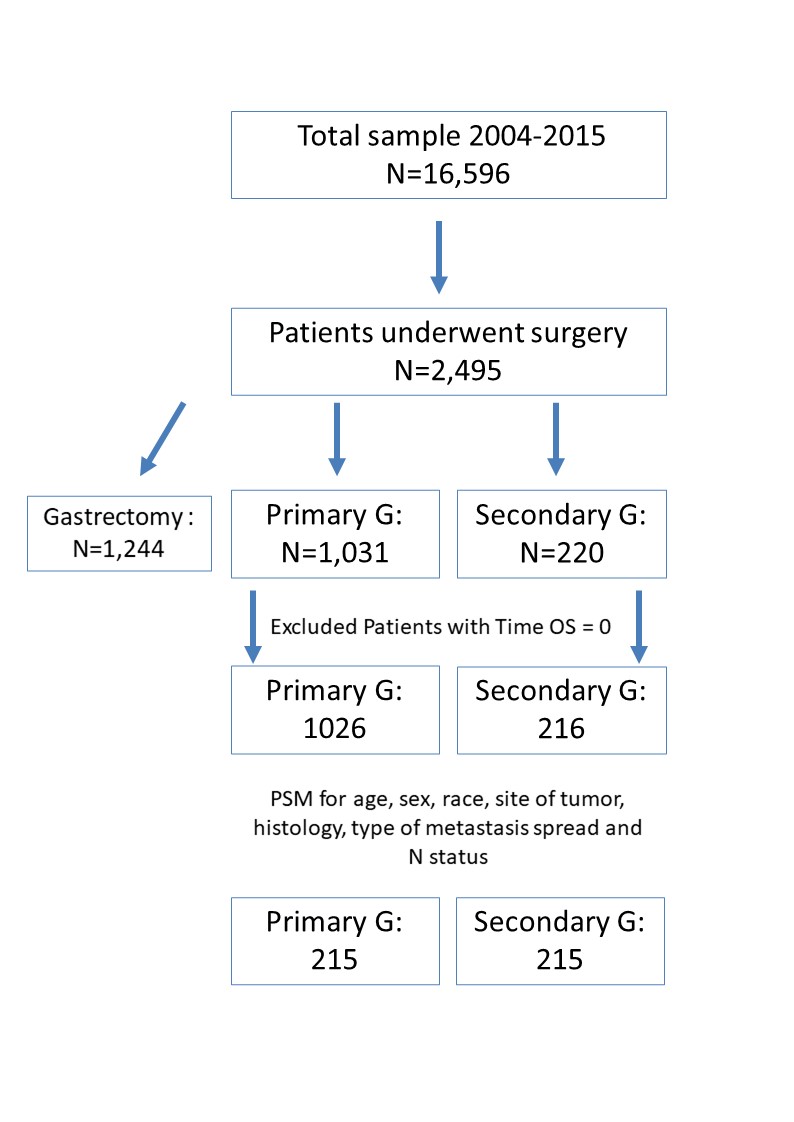


**eFigure1:** Flowchart of patient selection during the propensity score matched analysis of PG versus SG.


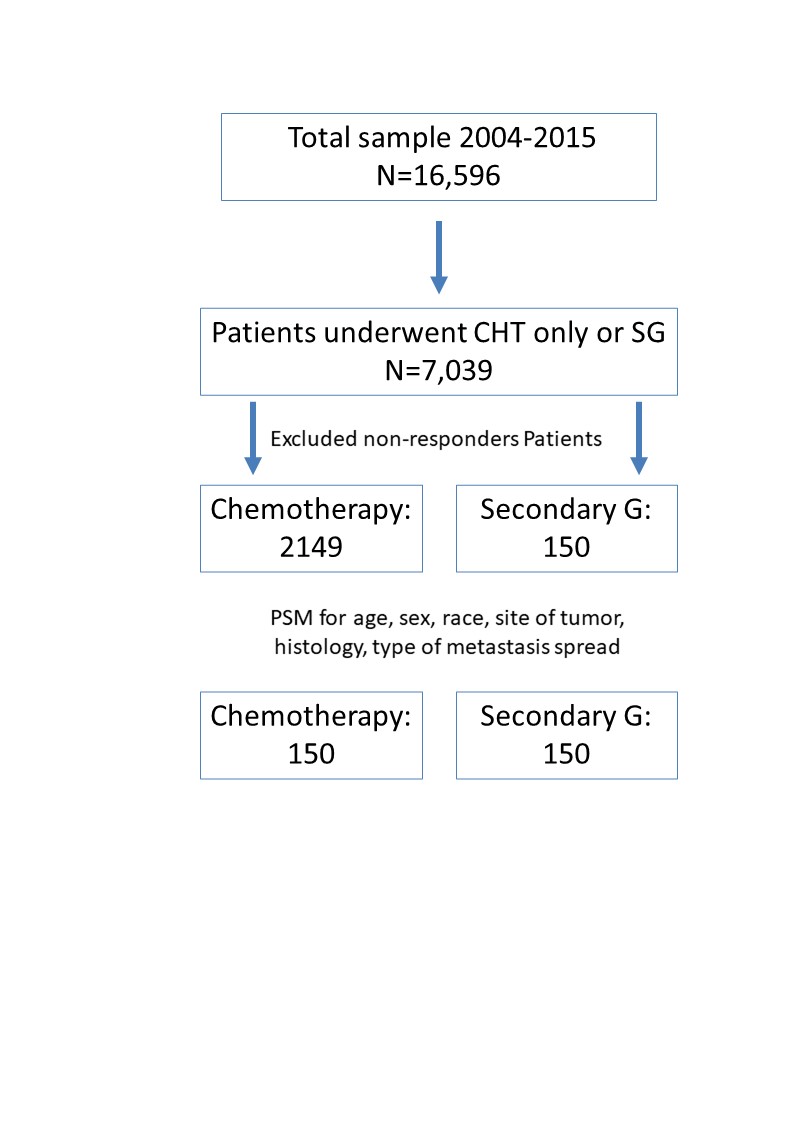


**eFigure2:** Flowchart of patient selection during the propensity score matched analysis of SG versus CHT.
